# Metabolic Profiles of New Unsymmetrical Bisacridine Antitumor Agents in Electrochemical and Enzymatic Noncellular Systems and in Tumor Cells

**DOI:** 10.3390/ph14040317

**Published:** 2021-04-01

**Authors:** Anna Mieszkowska, Anna M. Nowicka, Agata Kowalczyk, Agnieszka Potęga, Monika Pawłowska, Michał Kosno, Ewa Augustin, Zofia Mazerska

**Affiliations:** 1 Department of Pharmaceutical Technology and Biochemistry, Faculty of Chemistry, Gdańsk University of Technology, Narutowicza Str. 11/12, 80-233 Gdańsk, Poland; annnowak1@student.pg.edu.pl (A.M.); agnieszka.potega@pg.edu.pl (A.P.); monika.pawlowska@pg.edu.pl (M.P.); michal.kosno@pg.edu.pl (M.K.); ewa.augustin@pg.edu.pl (E.A.); 2 Faculty of Chemistry, University of Warsaw, Pasteura Str. 1, 02-093 Warsaw, Poland; anowicka@chem.uw.edu.pl (A.M.N.); akowalczyk@chem.uw.edu.pl (A.K.)

**Keywords:** antitumor unsymmetrical bisacridines, drugs in cell medium/extract, drugs under electrochemical transformations, FMO catalyzed metabolism, P450-mediated drug metabolism, UGT metabolic transformations

## Abstract

New unsymmetrical bisacridines (UAs) demonstrated high activity not only against a set of tumor cell lines but also against human tumor xenografts in nude mice. Representative UA compounds, named C-2028, C-2045 and C-2053, were characterized in respect to their physicochemical properties and the following studies aimed to elucidate the role of metabolic transformations in UAs action. We demonstrated with phase I and phase II enzymes in vitro and in tumors cells that: (i) metabolic products generated by cytochrome P450 (P450), flavin monooxygenase (FMO) and UDP-glucuronosyltransferase (UGT) isoenzymes in noncellular systems retained the compound’s dimeric structures, (ii) the main transformation pathway is the nitro group reduction with P450 isoenzymes and the metabolism to N-oxide derivative with FMO1, (iii), the selected UGT1 isoenzymes participated in the glucuronidation of one compound, C-2045, the hydroxy derivative. Metabolism in tumor cells, HCT-116 and HT-29, of normal and higher UGT1A10 expression, respectively, also resulted in the glucuronidation of only C-2045 and the specific distribution of all compounds between the cell medium and cell extract was demonstrated. Moreover, P4503A4 activity was inhibited by C-2045 and C-2053, whereas C-2028 affected UGT1A and UGT2B action. The above conclusions indicate the optimal strategy for the balance among antitumor therapeutic efficacy and drug resistance in the future antitumor therapy.

## 1. Introduction

Malignant tumors are the second most common cause of death after cardiovascular diseases. Although modern methods of cancer treatment and diagnosis have reduced patient mortality, some tumors, e.g., pancreatic, lung and colon cancers, are extremely resistant to chemotherapy [1,2]. Therefore, identification of the compounds that could offer significantly improved therapeutic efficacy against these diseases, especially those that are extremely complicated and insidious in nature, remains of great importance. Our group previously developed antitumor agents that reached an advanced stage of drug development. For example, imidazoacridinone C-1311 (Symadex^®^, Gdańsk, Poland) [3,4,5,6] was highly active against breast cancer in patients refractory to taxane treatment in phase II studies [7,8,9,10,11]. 9-Alkylamino-1-nitroacridines were also developed as antitumor therapeutic agents (Nitracrine^®^, Gdańsk, Poland) [12] and a new derivative of this group, compound C-1748, is under preclinical evaluation against prostate cancer [13,14,15]. C-1311 and C-1748 intercalate to DNA and following metabolic activation covalently crosslinks DNA strands [16,17]. At the cellular level, depending on the cell type and treatment conditions, both compounds trigger cell death via apoptosis, necrosis and also induce cellular senescence [18,19,20,21]. Additionally, C-1311 is a topoisomerase II inhibitor [22], selective FLT-3 kinase inhibitor [23] and effectively decreases tumor angiogenesis [24].

The following studies on the antitumor properties of acridines focused in other laboratories on the compounds constructed from two acridine moieties. A new class of the potent and highly selective antitumor agents was synthesized where the tetracyclic ring systems of two imidazoacridinones were tightly bound by *N*-methyl-dipropylamine-type linkers, UAs. [25]. Some of these compounds showed remarkably high activity and selectivity for colon cancer, including in vivo effects against human tumor HCT-116 xenografts in nude mice [26,27]. The synthesis and antitumor activities of novel bisacridines bearing new nonaromatic semirigid linkers between two 9-aminoacridines have also been described [28]. Importantly, the common features of these acridine dimers are their structural symmetry.

Recently our group synthesized and patented new promising bisacridines that were unsymmetrical being constructed from two different acridine monomers. They expressed activity not only against a set of 14 tumor cell lines, but also against 10 human tumor xenografts in nude mice and against Walker 256 adenocarcinoma in rats [29,30,31].

In the current studies, we intend to provide the background knowledge for our new antitumor unsymmetrical bisacridines, UAs. Compounds representative in their high antitumor activity were selected for these investigations (Table 1) [31]. They are different in the hydroxyl and methyl substituents in both acridine moieties and named C-2028, C-2045 and C-2053. We aimed firstly to know whether the metabolic products of the studied compounds retained their dimeric structure or whether they were transformed into monomers, imidazoacridinone and 9-amino-1-nitroacridine, which were studied earlier.

We started our research from the solubility evaluation of the potency of these compounds in respect to their acid-base equilibrium. Studies on the electrochemical transformations of these potent drugs were also performed. Drug metabolism was investigated with rat liver microsomes (RLMs) in the presence of enzyme cofactors for cytochrome P450 (P450), as well as with recombinant P450, flavin monooxygenase (FMO) and UDP-glucuronosyltransferase (UGT) isoenzymes. Furthermore, metabolic pathways of studied bisacridines occurring in the selected tumor cell lines with various expressions of UGT isoenzymes were demonstrated. We also investigated the impact of studied compounds on the metabolic enzyme activity considering that the drug deactivation and the subsequent drug excretion would be responsible for the intrinsic drug resistance in tumor cells [32,33,34].

Summing up, the prediction of the optimal strategy to achieve the appropriate balance between therapeutic efficacy and the adverse side effects, both resulting from P450, FMO and UGT-mediated metabolism, is very difficult. However, it is crucial for designing new unsymmetrical bisacridines as effective antitumor therapeutics described herein.

## 2. Results

The three UAs synthesized in our laboratory [29,30,31], named C-2028, C-2045 and C-2053, are subjected to the current studies and their structures are presented in Table 1. In comparison to C-2028, C-2045 possesses a hydroxyl group in the imidazoacridinone ring and an additional methyl group in the acridine part. In contrast, compound C-2053 was explored to elucidate the lack of the hydroxyl group, whereas the additional methyl group in the acridine ring was still present in the 1-nitroacridine structure.

### 2.1. Physicochemical Properties

The antitumor activity of the potent drugs results not only from their cytotoxicity against tumor cells, but also from their properties, which are responsible for the drug transport and drug delivery to the specific molecular target. For example, the parameters of acid-base equilibrium determine the rate of the protonated form of the drug (more polar and more soluble in water than the non-protonated form) in the stomach, intestines or liver blood. On the other hand, the susceptibility to drug metabolism can be simulated by the ability to undergo redox transformation under electrochemical conditions.

The solubility of investigated compounds was tested to select the best overall solute for subsequent studies of biological activity under different conditions. We demonstrated that all new compounds were soluble in redistilled water, ethanol and DMSO up to 10 mM. The compounds were also easily soluble in 0.05 mM ammonium formate buffer (pH 3.4), which is the main eluent applied in HPLC analyses. However, the studied compounds demonstrated lower solubility (1 mM) in buffer solutions at pH 7.4, including 0.1 M potassium phosphate, 0.05 M Tris-base and 0.1 M HEPES buffers, which are often applied in experiments related to metabolic transformations of the drugs. Therefore, the following three-step solution procedure was proposed for the subsequent studies on drug metabolism: (i) 10 mM solution of UAs in DMSO, (ii) dilution of the DMSO solution in HEPES buffer (pH 7.4) to yield a 1 mM stock solution and (iii) the preparation of final solutions of the UAs at 0.05 or 0.1 mM in HEPES buffer (pH 7.4), which were applied directly for drug incubation studies.

**Figure 1:**
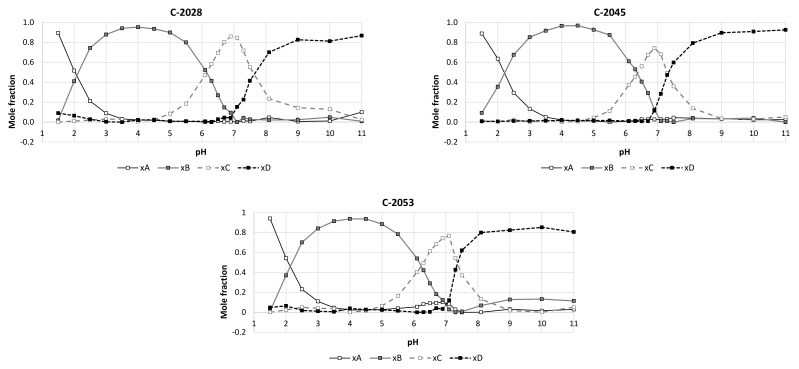
The titration curves obtained by the principal component analysis for the studied compounds as the relations between mole fractions of each spectral form in UV-vis spectra and pH values of the compound solutions. Individual spectral forms: xA, xB, xC and xD correspond to different degrees of bisacridine protonation, H_3_UA^+3^, H_2_UA^+2^, HUA^+^ and UA. The pH values equal to 0.5 mole fraction for adjacent forms respond to pKa values specific for each acid-base equilibrium.

The acid-base properties of the studied compounds were determined by spectrophotometric titration. The results of the experiments and calculations are demonstrated in Figure 1. The principal component analysis for UV-vis spectra at pH range from 1.5 to 11.0 indicated that studied compounds (C-2028, C-2045 and C-2053) exist in four differently protonated forms. The pKa values presented in Table 2 indicated the pH points, which correspond to acid-base equilibria.

**Figure 2:**
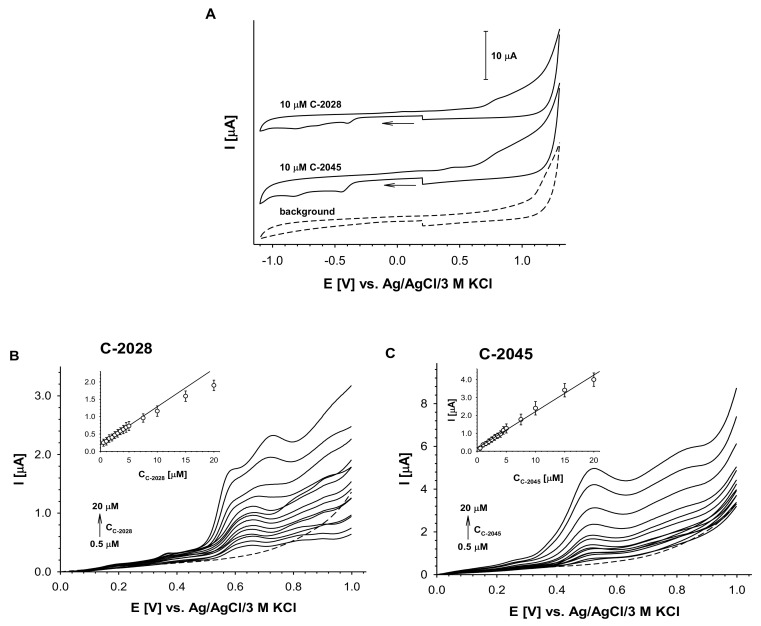
Electrochemical analysis of C-2028 and C-2045 compounds. (**A**) Cyclic voltammograms of two UAs compounds (cUAs = 10 µM) in comparison to the background. (**B**) C-2028 and (**C**) C-2045 DP voltammograms. The solutions were prepared at least 1 h before measurements. Insets in B and C: dependence of DPV peak current intensity vs. UAs concentration. Experimental conditions: cUAs = 0.5–20 µM, 0.02 M borate buffer with 0.15 M NaCl, pH 7.4, glassy carbon electrode (*ϕ* = 3 mm), scan rate: 100 mV/s, pulse time 0.01 s, interval time 0.1 s.

The voltammetric activities of C-2028 and C-2045, illustrated in Figure 2A, were observed in the positive and negative potential regions. The signal at the positive potential resulted from the oxidation of the imidazoacridinone ring in the dimer structure and the second from the reduction of a 1-nitroacridine moiety. The absence of the reverse cathodic peaks in the positive potential range and of the reverse anodic peaks in the negative potential range indicated that the observed electrochemical reactions of UAs are irreversible processes. This irreversibility is probably due to the strong tendency of electrochemical products to adsorb onto the electrode surface, although each voltammetric measurement was carried out with freshly cleaned electrodes.

To minimize the effects of electrode absorption processes on the current signals, differential pulse voltammetry (DPV) was applied in the positive potential range. Representative DP voltammograms for both compounds on a glassy carbon electrode are presented in Figure 2B,C. The first voltammogram of C-2028 exhibited double signal at a potential from 0.55 V to 0.70 V and this transite into one signal at concentrations higher than 7.5 µM. This behavior was not observed in the case of C-2045, where only one anodic signal at a potential of approximately 0.52 V was observed over the entire studied concentration range (0.5–20 µM). The linear dependency, I = f (cUAs), confirmed that the obtained anodic current signals can be used for analytical purposes; however, the signal of C-2028 was linear over a narrower concentration range than in the case of C-2045.

### 2.2. Transformations of the Compounds in Noncellular Systems

The crucial question regarding the observed antitumor activity of the studied UAs [29,30,31] was whether the observed high potency of the compounds is a specific, characteristic feature of the dimeric structure or whether it is the sum of monomer activities, which would be observed after metabolism of the UA dimers. Considering the above, the subject of the following studies relates to the possible transformations of these compounds, first in the selected noncellular systems and then in cancer cells. Therefore, the transformation studies with noncellular systems are presented below and include several aspects of drug metabolism: (i) the role of P450 inducers in drug transformations with rat liver microsomal, RLM, enzymes, (ii) the phase I metabolic potency of the selected human recombinant P4503A4/2C19 and FMO isoenzymes and (iii) the transformation role of phase II UGT isoenzymes The products of the investigated metabolic transformations will be compared with those of the electrochemical conversion and their structures will be identified.

#### 2.2.1. Preliminary Research on Metabolic Transformations of UAs with RLMs

The initial studies on drug metabolism of new UAs were performed with rat liver microsomes under the optimal conditions. HEPES buffer (0.1 M) at pH 7.4 with 2 mM NADPH and 0.05 mM UAs (C-2028 and C-2045) were selected.

**Figure 3:**
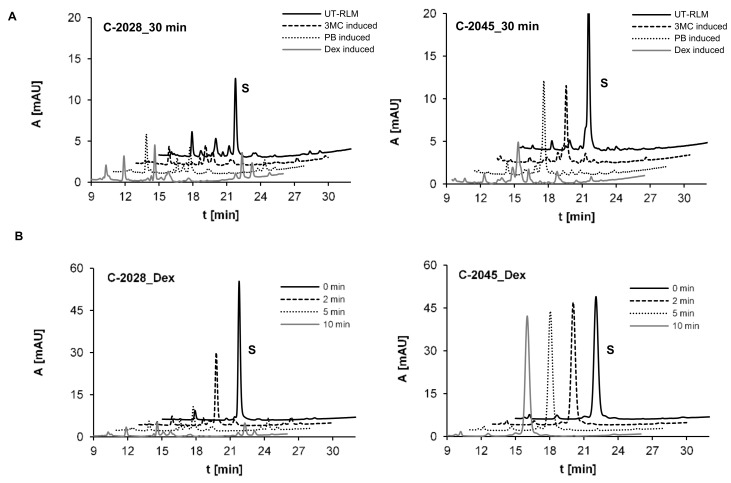
HPLC analysis of the reaction mixture after 30 min incubations of 0.05 mM compounds with (**A**) UT-RLMs and P450-RLMs (2 mg/mL) induced with 3-methylcholanthrene (3MC), phenobarbital (PB) and dexamethasone (Dex) and (**B**) after 2, 5 and 10-min compound incubation with P450-RLM, Dex-induced. The 2 mM NADPH was applied as the cofactor. The solid, dotted and gray lines representing the same retention times are offset for better visualization.

Metabolic transformations of C-2028 and C-2045 were investigated with untreated rat liver microsomes, UT-RLMs and with P450-RLMs obtained after P450 induction with 3-methylcholanthrene (3MC), phenobarbital (PB) and dexamethasone (Dex). The results of HPLC analysis presented in Figure 3A indicated that both UAs were intensively transformed with RLMs into a large number of metabolites. The higher conversion rates of 100% and 80% for C-2028 and C-2045, respectively, were observed for Dex-induced P450-RLMs. Figure 3B shows the analogous results acquired over a shorter period: 10 min. Compound C-2028 was completely consumed after 10 min, yielded at least four metabolites at the retention times (t_R_ values) of 14.63, 14.88, 15.17 and 22.36 min, whereas C-2045 was not metabolized under these conditions.

#### 2.2.2. Metabolism of UAs with Human Recombinant P4503A4 and P4502C19 Isoenzymes

**Figure 4:**
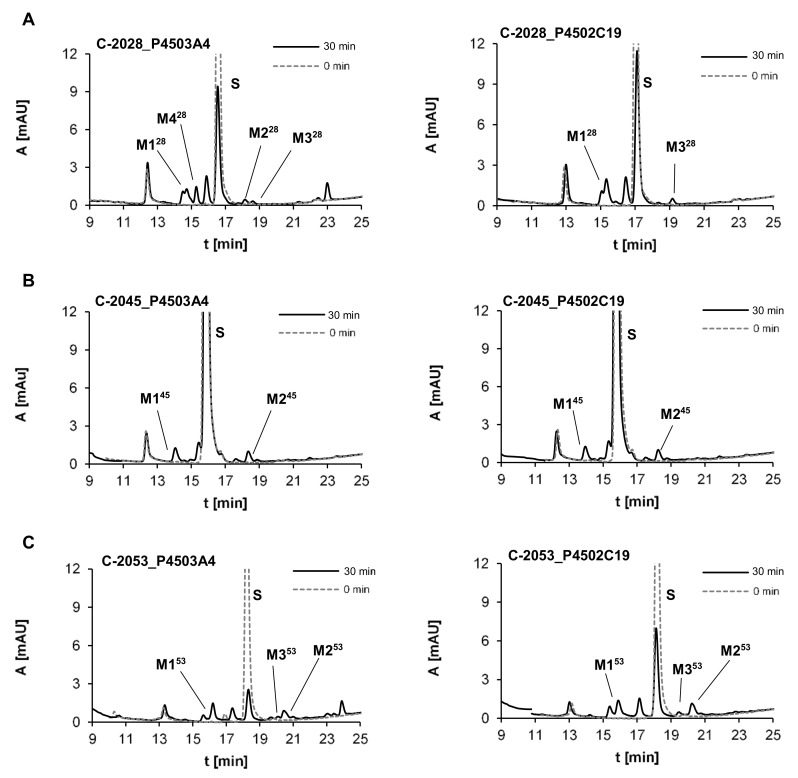
HPLC analysis of the reaction mixture after 30 min incubations of 0.05 mM (**A**) C-2028, (**B**) C-2045 and (**C**) C-2053 with 1 mg/mL of cytochromes P4503A4 and P4502C19. The 2 mM NADPH was applied as the cofactor.

P4503A4 is generally expressed in the liver and is also specifically concentrated in the coronary artery and cerebellum, whereas P4502C19 is expressed in the bone marrow and small intestine [35]. P4503A4 was selected for the present study because it is responsible for the metabolism of nearly 50% of drugs [36] and the majority of antitumor therapeutic agents [37,38]. In contrast to the P4503A4 subtype, the P4502C19 isoenzyme is responsible for the transformation of only 15% of known pharmaceuticals with narrow therapeutic windows. However, we selected this isoenzyme because it metabolizes the group of drugs that are frequently encountered in therapy as the antiplatelet warfarin and clopidogrel, the antiepileptic carbamazepine and the proton pump inhibitor omeprazole [39,40]. Considering the presence of an aminoalkyl side chain in the structures of studied UA compounds, we also noticed that P4502C19 is strongly involved in the metabolism of this type of amino group in other therapeutics, including the antidepressant imipramine [41]. Figure 4 presents the chromatograms obtained after the incubation of the three studied compounds, C-2028, C-2045 and C-2053, with human recombinant isoenzymes P4503A4 and P4502C19.

**Figure 5:**
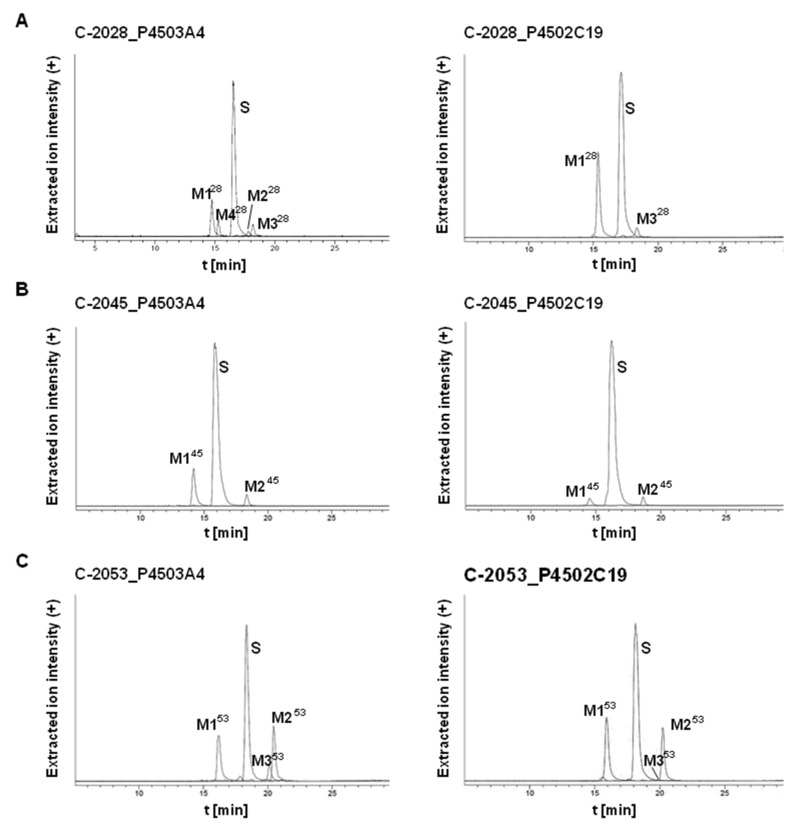
Extracted ion chromatograms of HPLC-MS analysis for all metabolites obtained after 30-min incubations of 0.05 mM (**A**) C-2028, (**B**) C-2045 and (**C**) C-2053 with 1 mg/mL of P4503A4 and P4502C19 isoenzymes. The 2 mM NADPH was applied as the cofactor.

These data were improved by the extracted ion chromatograms in Figure 5. Two or more metabolites were observed for each compound and the rate of metabolism was different among them. We found that 72% and 59% of C-2028, was transformed in the presence of P4503A4 and P4502C19, respectively, whereas C-2045 was transformed much more slowly, with 30% and 11% of the substrate consumed after 30 min. The highest rate of metabolism was observed for C-2053, which possesses the methyl group in the acridine ring and lacks a hydroxyl substituent in the imidazoacridinone monomer. The rates for it in the presence of P4503A4 and P4502C19 after 30 min were approximately 77% and 64%, respectively.

The metabolism of the studied compounds with recombinant P4503A4 and P4502C19 isoenzymes was analyzed by HPLC with ESI-MS detection. Table 3 presents: the retention times (t_R_) and the *m*/*z* values found at t_R_ for the metabolites by ESI-MS and ESI-Q-TOF-MS. The obtained results indicated that all three compounds, C-2028, C-2045 and C-2053, were transformed into two main stable products. The first one, M1^X^ (where x = 28, 45 or 53), had a shorter t_R_ than the substrate and was characterized by the *m*/*z* of M^X^-30, whereas the second metabolite, M2^X^, had a longer t_R_ than the substrate and the *m*/*z* of M^X^-18. For two of the studied drugs, C-2028 and C-2053, M3^X^ metabolites with an *m*/*z* equal to M^X^-14 were also observed. It should be underlined that all products of transformations mentioned above retained their *m*/*z* values, which reflected the retention of the dimer structure of the metabolites. Only the M4 product for C-2028 (M4^28^) is suggested to be the acridine monomer. The studies on the structure of metabolites were improved with the aid of electrochemical transformations of the drugs presented below.

#### 2.2.3. Results of Electrochemical Simulations of Drug Metabolism

**Figure 6:**
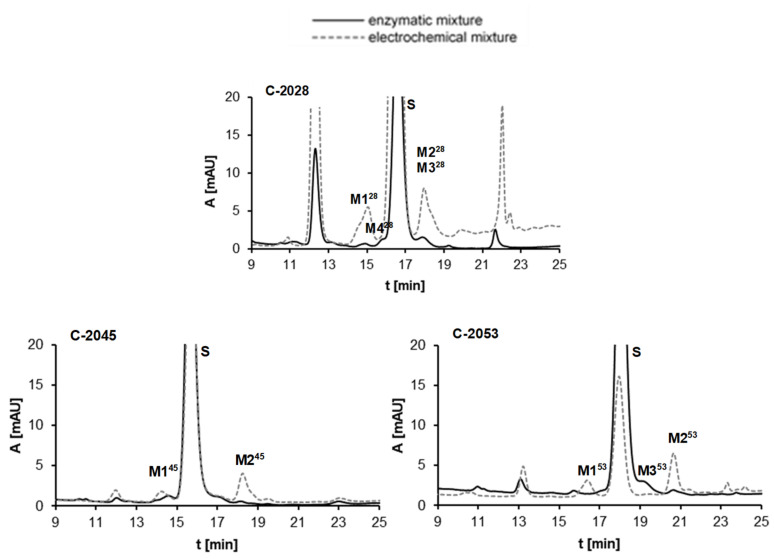
Comparison of HPLC analyses of the reaction mixtures after 30 min enzymatic (UT-RLMs) incubations and the electrochemical conversions of 0.05 mM C-2028, C-2045 and C-2053. Experimental conditions: enzymatic analysis—0.1 M HEPES buffer, pH 7.4, 2 mg/mL UT-RLMs, 2 mM NADPH, 2 mM MgCl_2_; electrochemical analysis—water-methanol (1:1, *v*/*v*) with 0.1% formic acid as electrolyte, potential range −2.5–0 V, glassy carbon working electrode (*ϕ* = 8 mm).

The voltammetry analysis presented above (Figure 2) indicated the possibility of electrochemical transformations of the studied compounds. Considering the relations between electrochemical and enzymatic transformations of acridine derivatives investigated earlier [42,43], we performed the electrochemical conversion of the studied compounds C-2028, C-2045 and C-2053 in the potential range −2.5–0 V according to the new method developed in our group [44]. The reaction mixture was analyzed by HPLC under conditions identical to those applied above for metabolic transformations with RLMs. Figure 6 presents the comparison of representative chromatograms obtained after liver microsomal incubation and electrochemical conversion of the UAs investigated. The results observed for M1–M4 metabolites of C-2028, M1-M2 of C-2045 and M1–M3 of C-2053 after in vitro enzymatic transformations expressed good agreement (in terms of *m*/*z* values, retention times and UV-vis spectra) with those produced in an electrochemical system. Detailed tandem mass spectrometry (MS/MS) of the products generated electrochemically [45] supported the proposed tentative structures of UA metabolites presented in the last column of Table 3 and described below.

It was demonstrated that the metabolism of all three studied compounds occurred on the nitro group in the acridine ring. Two main products of the transformations were found for each drug. For the first ones, M1^28^, M1^45^ and M1^53^, 1-amino substituent was in the acridine ring, whereas the second group of metabolites, M2^28^, M2^45^ and M2^53^, have an additional pyrazole ring between positions 1- and 9- of acridine with one nitrogen atom oxidized. Considering the third group of metabolites, M3^28^ and M3^53^, we determined the demethylation products on the nitrogen atom in the linker chain. The proposed that metabolite structures were also supported by our previous results obtained after P450-mediated metabolism of the monomer compounds 9-amino-1-nitroacridine [34,46,47] and imidazoacridinone [48,49].

#### 2.2.4. Metabolism of UAs with Human Recombinant Flavin Monooxygenases, FMO

FMO-mediated catalysis gives the polar, readily excreted metabolites and relates particularly to these structures, which possess the aliphatic amino groups. These enzymes are oxidoreductases with the potential for the development as biocatalysts in biotechnological processes as well as in drug metabolism [50,51]. There are several reports about the participation of FMO isoenzymes in the detoxification of some antitumor agents Contrary to other catalysators of drug metabolism, FMOs are rather resistant for the action of enzyme activity modulators [52,53]. 

**Figure 7:**
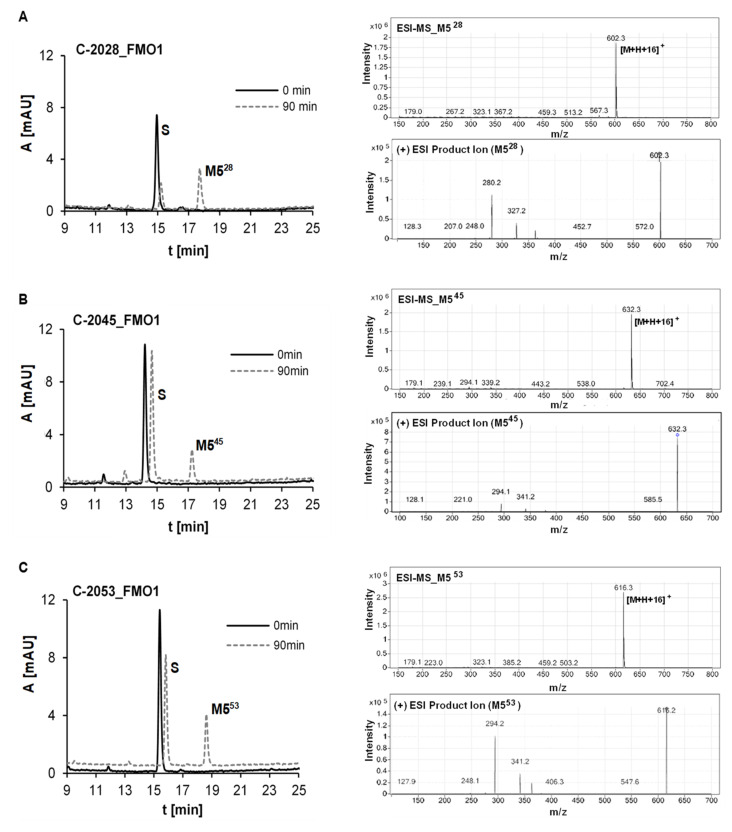

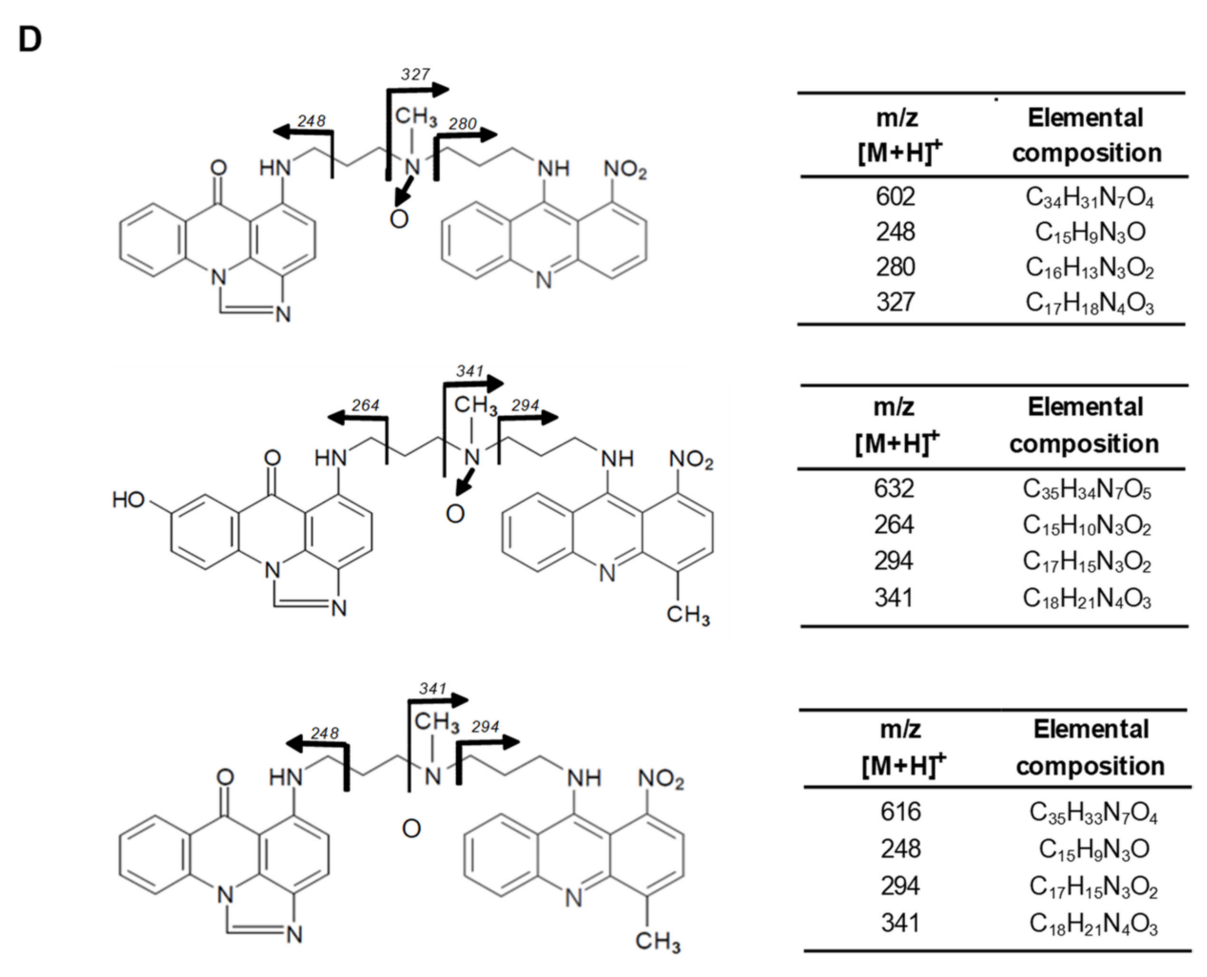
HPLC, ESI-MS and ESI-MS/MS analysis of the reaction mixture after 90 min incubations of 0.01 mM (**A**) C-2028, (**B**) C-2045 and (**C**) C-2053 with 0.5 mg/mL of FMO1. 0.3 mM NADPH was applied as the cofactor; (**D**) structure identification.

In the search for phase I enzymes involved in metabolism of UAs, other than cytochromes P450, three forms of human recombinant flavin monooxygenases (FMO1, FMO3 and FMO5) were incubated with C-2028, C-2045 and C-2053. Figure 7A–C demonstrated high transformation rates after 90 min incubation with FMO1 for all compounds. In contrast, FMO3 and FMO5 were not active toward all tested UAs. Metabolism with FMO1 yielded one main metabolite, M5, for each compound. ESI-MS analyses of M5^28^, M5^45^ and M5^53^ indicated a product 16 *m*/*z* units higher than the substrate. Moreover, ESI-MS/MS ion spectra of metabolites M5^X^ showed in Figure 7D that all chosen precursor ions derived from these metabolites break down into three characteristic mass ions. One of them indicated the addition of the oxygen atom as a result of the compound incubation with FMO1. Therefore, it was assumed that the obtained metabolites M5^X^ are N-oxides of C-2028, C-2045 and C-2053. The proposed metabolite structures were also supported by our previous results obtained after FMO mediated metabolism of the imidazoacridinone monomer [48,49].

#### 2.2.5. Glucuronidation of UAs with Human Recombinant UGT Isoenzymes

UGT families catalyze nearly 35% of phase II metabolic transformations with UGT2B7 and 1A1 in the highest proportions [54,55,56]. In the liver, glucuronidation mainly occurs with UGT2B4, whereas in the stomach, UGT1A6, UGT2B15 and UGT2B17 are involved. UGT1A6, UGT1A9 and UGT2B7 were specifically identified in the kidney and the analysis of urine allows the identification of drug and narcotic glucuronides [57,58]. The specific feature of UGT1A9 allows for the glucuronidation of not only oxygen atoms but also nitrogen and even nucleophilic carbon atoms [59,60]. On the other hand, UGT1A10 is expressed generally outside the liver, particularly in colon tissue [61]. Therefore, UGTs in the colon, where the drug bioavailability is usually limited after p.o. drug delivery, would participate in the first step of metabolic transformations.

A screening of the UGT-mediated metabolism of the studied compounds was performed here with human recombinant isoenzymes of the UGT1A and UGT2B families listed in Table 4.

**Figure 8:**
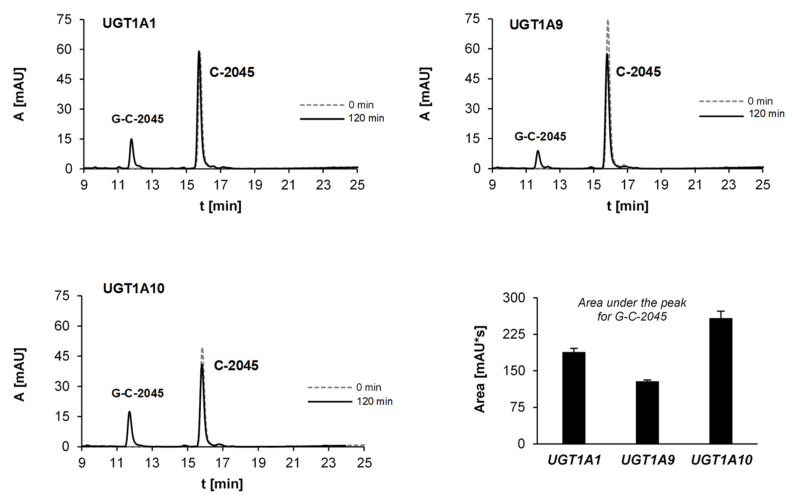
HPLC analysis of the reaction mixtures after 120-min incubations of 0.05 mM C-2045 with the selected recombinant isoenzymes of UGT1A family: UGT1A1, UGT1A9 and UGT1A10 (1 mg/mL) and 5 mM UDPGA as the cofactor. The comparison of the related glucuronide peak areas.

The set included the recombinant human UGT1A isoforms 1A1, 1A6, 1A9 and 1A10, expressed in Sf9 cells as His tag proteins and four human recombinant UGT2B isoforms 2B4, 2B7, 2B15 and 2B17, which were all incubated with UDPGA as a cofactor. The obtained results strongly indicated that only C-2045 underwent glucuronidation and only with isoenzymes of the UGT1A family (Table 4). Figure 8 demonstrated that UGT1A10 metabolized this drug with the highest rate (18% after 120 min) The observed HPLC peak was analyzed by UV-vis and ESI-MS techniques (Table 5).

The ESI-MS spectrum of C-2045 metabolites indicated a product 176 *m*/*z* units higher than the substrate, which strongly indicates the presence of glucuronic acid residue in the metabolite. Moreover, the results of ESI-MS analysis for the product, giving the *m*/*z* equal to 792.25, indicated that this metabolite retained dimeric structure of the substrate. After hydrolysis of the glucuronide metabolite, the presence of an ion with the *m*/*z* of 616.25 (792.25–176) confirmed that C-2045 underwent glucuronidation and the metabolite remained dimeric.

### 2.3. Metabolism of Studied UAs in Colon Tumor Cells

The studies on the enzymatic transformations of UAs performed above demonstrated their high ability to metabolize with enzymatic fractions of rat liver and lower metabolic potency in the presence of P4503A4 and P4502C19 isoenzymes. Particularly, one can see that compound C-2045 selectively underwent metabolism with UGT isoenzymes. Therefore, our studies on drug metabolism in tumor cells considered two aspects. First, we intended to compare the contents of the incubation media obtained after this compound treatment for three different colon tumor cell lines: (i) HCT-116, which is naturally characterized by a low level of UGT expression; (ii) HCT-116 stably transfected with the UGT1A10 gene and (iii) HT-29, with naturally high expression of UGT enzymes. On the other hand, we considered that the glucuronide derivatives of the drugs or other xenobiotics are usually detoxification products and they can be excreted outside the cells. Therefore, following incubation with the studied antitumor agents, we analyzed not only the content of the cell extract but also the content of the cell incubation medium. The tumor cells were treated by the compound concentrations, which are the multiples of IC_50_ determined earlier for HCT-116 [62].

#### 2.3.1. Transformations of C-2045 in Colon Cell Lines with Different UGT Expression

**Figure 9:**
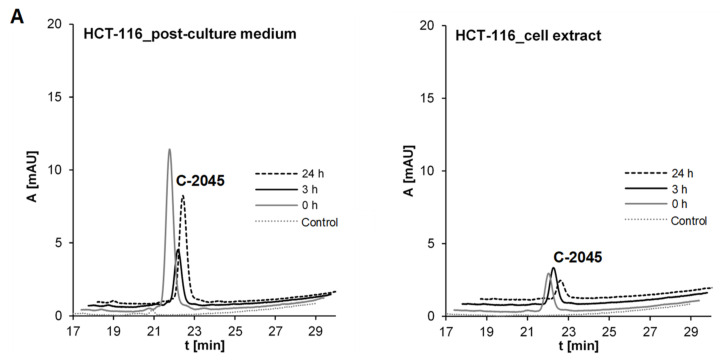

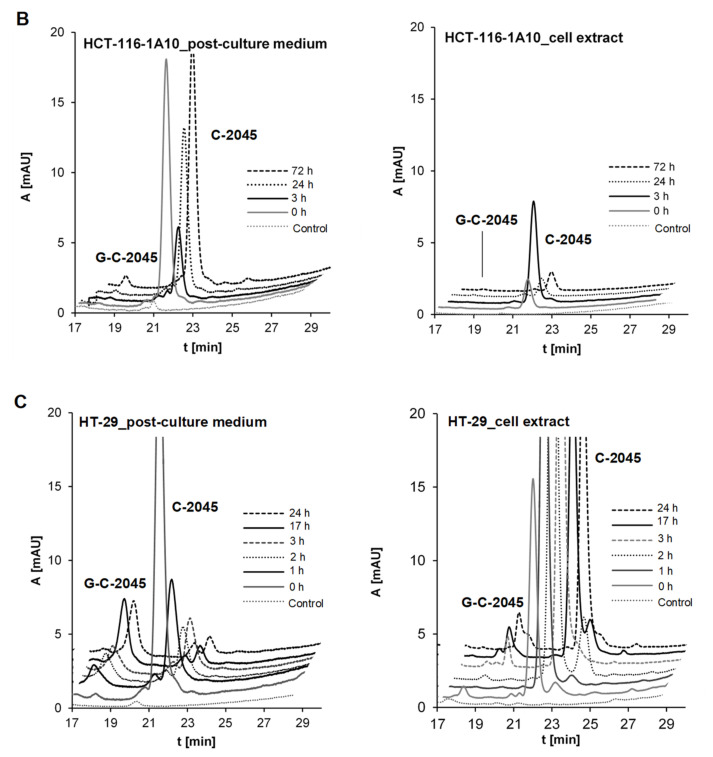
Glucuronidation of C-2045 in (**A**) HCT-116 cells, (**B**) HCT-116-UGT1A10 cells and (**C**) HT-29 cells. Compound concentrations on the plate were: 50 μM, 50 μM and 25 μM, respectively. The results of HPLC analyses of post-culture media and cell extracts are presented as a set of chromatograms: Control (control of medium/cell extract from cells not treated with the compound) and after desired incubation times. Analysis at 430 nm by RP-HPLC (Shimadzu) and ESI-MS.

The three above-described cell lines were treated with the C-2045 compound, which is the UA derivative with a hydroxyl group in the imidazoacridinone ring (Table 1). The obtained results are presented in Figure 9A. HPLC analysis of HCT-116 tumor cell medium indicated a fast decrease in the peak height after 3 h and a following increase after 24 h. In comparison, the cell extract contained a rather low concentration of the compound after 3 h and a much lower concentration after a longer time, i.e., 24 h of incubation. These results indicated that the studied drug first penetrated well HCT-116 cells in the first hours after drug additions and after 24 h was in part eliminated into the cell medium. No metabolite products were observed.

The incubation of HCT-116-1A10 cells with the C-2045 compound (Figure 9B) resulted in its lower cell medium concentration after 3 h and a return after 24 h to the high level comparable to that of the starting incubation point. Moreover, a new HPLC band of low intensity with a t_R_ close to 18 min was identified in the cell medium after 24 and 72 h. This band was not visible in the cell extract, where the highest concentration of the substrate was observed after 3 h and diminished after 24 h and longer. Therefore, HCT-116 cells overexpressing UGT1A10 metabolized the substrate to the derivative, which was found in the cell medium rather than in the cell extract.

In the case of HT-29 tumor colon cells characterized by the natural expression of UGT1A10 and some other UGT isoenzymes [63] high intensity of the HPLC peak substrate in the cell medium was observed only at the beginning of the experiment and it strongly decreased after 3 h and longer periods of incubation (Figure 9C). In this case, the significant concentration of the new band of t_R_ near 18 min was demonstrated. On the other hand, the cell extract of HT-29 contained a much higher concentration of the substrate after 3, 17 and 24 h than did that of HCT-116-1A10 cells. Therefore, HT-29 cells with natural expression of UGT1A10 allowed for a higher concentration of the drug in the cell extract than in the incubation medium after a longer incubation time.

**Figure 10:**
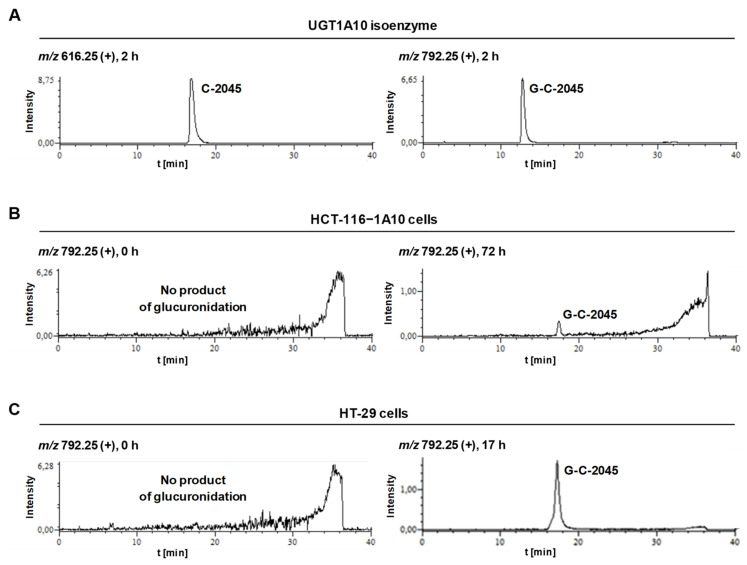
Representative extracted ion chromatograms of HPLC-MS analysis acquired 616.25 *m*/*z* for C-2045 and 792.25 *m*/*z* for the sample obtained by incubation of C-2045 with (**A**) isoenzyme UGT1A10, (**B**) the cells HCT-116-1A10 and (**C**) the cells HT-29 after positive ionization.

The HPLC bands obtained for HCT-116-1A10 and HT-29 cells after incubation with the C-2045 compound (Figure 9B,C) were analyzed by ESI mass spectrometry in positive ion mode with a single quadrupole and the results shown in Figure 10 indicated the presence of mass ions with the *m*/*z* of 792.25. These values were identical to the *m*/*z* value presented above for the UGT1A10 glucuronidation product found in noncellular medium (Table 5). In conclusion, the tumor cell metabolite of C-2045 was identified as the glucuronide, G-C-2045, probably on the hydroxyl group of the imidazoacridinone ring of the dimer structure.

#### 2.3.2. Metabolism of UAs in the HT-29 Tumor Cell Line of High UGT Expression

**Figure 11:**
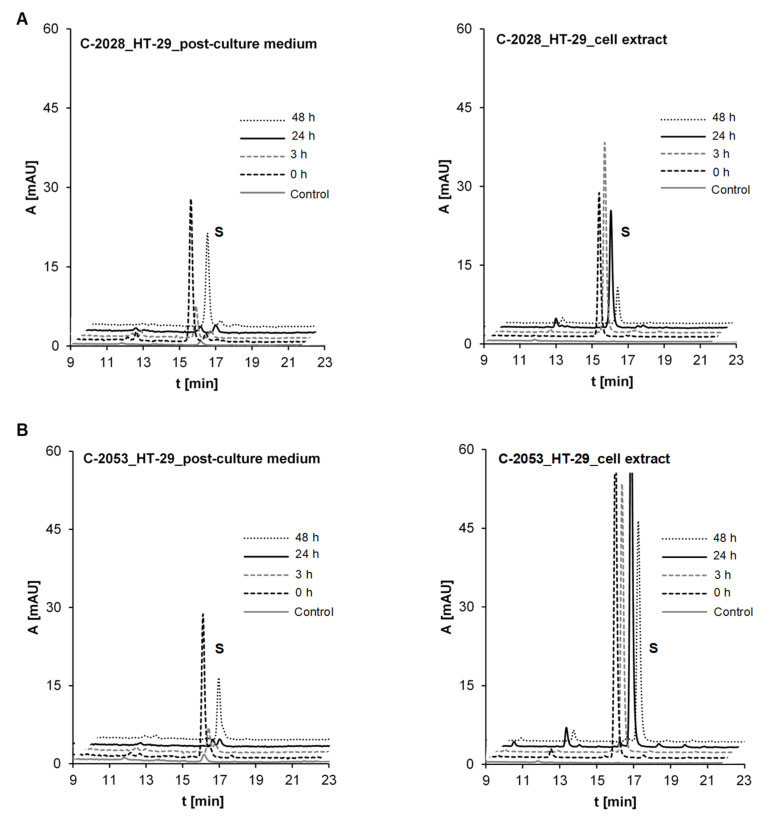
Kinetic of drug transfer between culture medium and cell extract of (**A**) C-2028 and (**B**) C-2053 in HT-29 cells. Compound concentration on the plate was 5 μM. The results of HPLC analyses of post-culture media and cell extracts for: control of medium/cell extract from cells not treated and after desired incubation times. Analysis at 430 nm by RP-HPLC (Waters).

HT-29 cells were selected for the comparison of metabolism observed for the unsymmetrical dimer compounds, C-2028 and C-2053, which did not possess the hydroxyl group in the imidazoacridinone ring (Table 1). HPLC analysis of the post-culture media and cell extracts after incubation with these compounds (Figure 11) did not indicate the presence of metabolites. Therefore, the kinetic of drug transfer between the culture medium and the cell extract was observed in the next step of studies.

Firstly, there was observed that the penetration between the incubation medium and the cell exhibited different kinetics for each compound. In the case of non-substituted C-2028, the concentration in the cell medium decreased after 3 hours of incubation and after 48 hours, the compound returned to the cell medium. Specifically, the drug concentration in the cell extract was high up to 24 hours and diminished significantly after 48 hours. In the case of C-2053, with the 4-methyl group in the 1-nitroacridine ring, a rather low concentration of the substrate was observed in the cell medium. However, an extremely high level of C-2053 was found in the cell extracts after drug incubation. It seems that this compound is delivered quickly into the cell and thereafter, up to 24 hours, its level increases and only slightly decreases after the next 24 hours.

### 2.4. The Compound’s Impact on P450 and UGT Isoenzymes Activity

The low level of compounds transformations with P4503A4 and P4502C19 isoenzymes and the lack of metabolic products for some of UGT1A and UGT2B isoenzymes were demonstrated above. In addition, in tumor cells the metabolic products were observed only for C-2045. This was the reason we directed following studies towards the potential of studied compounds to change enzymatic activity of drug metabolizing P450 and UGT isoenzymes. We considered that the activity modulation of studied enzymes, which catalyze not only xenobiotic metabolism, but also metabolism of natural substrates in patient organism will have significant impact on the final effectiveness of the applied therapy procedures.

#### 2.4.1. P4503A4 Inhibition by C-2045 and C-2053

**Figure 12:**
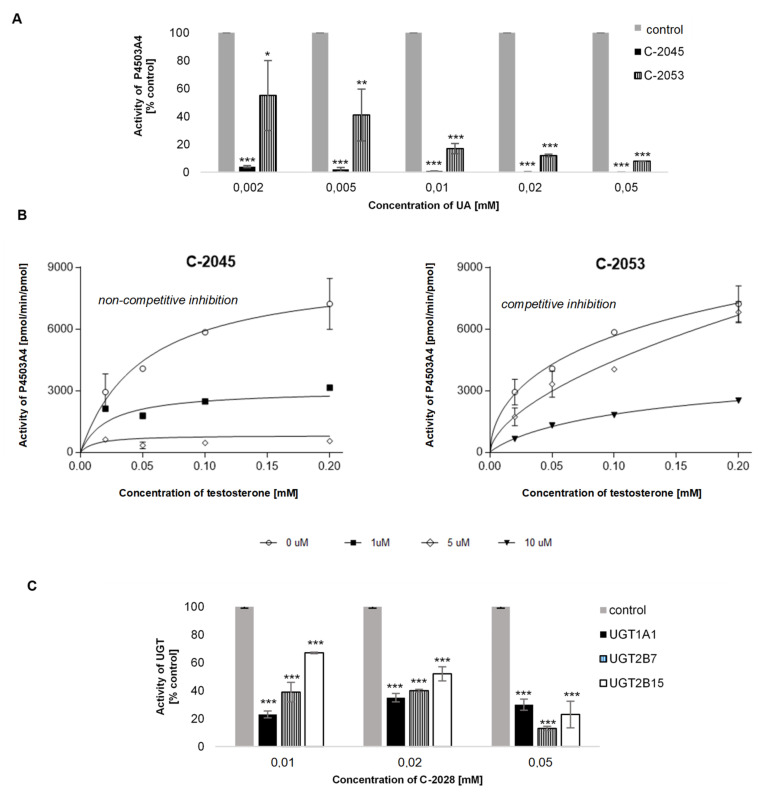
The effect of UAs concentration on P4503A4 and UGT activity. The modulation of: (**A,B**) testosterone β-hydroxylation with P4503A4 by C-2045 and C-2053 (**C**) glucuronidation activity of human recombinant UGT isoenzymes studied with the specific substrates: SN-38 for UGT1A1, epirubicin for UGT2B7 and TFK for UGT2B15 by C-2028. Values are expressed as the mean ± SD of duplicate experiments. Enzymatic activities towards standard substrates in the absence of UAs were normalized to 100%. Comparisons were made using one-way ANOVA, followed by Bonferroni’s multiple comparisons test, or an unpaired *t*-test. Levels were considered significant at * *p* < 0.05; ** *p* < 0.01; *** *p* < 0.001.

The obtained results (Figure 12A) showed that two tested UAs: C-2045 and C-2053, inhibited the activity of P4503A4 isoenzyme. The compound with the hydroxyl group in the imidazoacridinone ring, C-2045, showed over 90% decrease in activity of P4503A4 at all concentrations. Meanwhile the compound with methyl group in 1-nitroacridine ring, C-2053, inhibited the activity of P4503A4 above 50% for all concentrations. Kinetic analysis was performed for further characteristic of the enzyme inhibition induced by both compounds. Testosterone β-hydroxylation performed in the presence of various concentrations of studied compounds and analyzed by fitting the data to nonlinear regression model (Figure 12B) revealed that C-2045 inhibited activity of human recombinant P4503A4 isoenzyme according to non-competitive and C-2053 according to competitive mechanism.

#### 2.4.2. UGT Isoenzymes Inhibition by Bisacridines Studied

The results at Figure 12C demonstrated that studied compounds induced significant inhibition of the selected isoenzymes UGT1A1, UGT2B7 and UGT2B15. For example, at low concentrations of C-2028 the activity decrease of the UGT1A1 isoenzyme reaches even 77%. In contrast, at higher concentrations of this compound, the inhibition rate is slightly lower, 65–70%. Thus, with concentration increase of C-2028, no increase in the inhibition rate of UGT1A1 isoenzyme is observed. While 0.01 mM and 0.02 mM concentration of C-2028 caused a 60% decrease in the activity of UGT2B7 90% inhibition occurred in the presence of the highest concentration 0.05 mM. Similarly, as compound concentration increased, the degree of activity inhibition of UGT2B15 significantly increased. At the lowest concentrations, the inhibition was approximately 30% and at the highest as much as 80%.

## 3. Discussion

Among more than 50 unsymmetrical dimeric acridine derivatives, the selected compounds synthesized by our group were active not only against the set of 14 tumor cell lines but also against more than 10 human tumor xenografts in nude mice and Walker 256 adenocarcinoma in rats [29,30,31]. The representative compounds with the highest antitumor potency, C-2028, C-2045 and C-2053, were the subject of the studies presented herein. We demonstrated the physicochemical characteristics, investigated the enzymatic activities and performed metabolic transformations in tumor cells of these antitumor agents to predict their behavior during the following patient treatments.

The pKa values, shown in Table 2, indicated that the second and third acid-base equilibria (pKa values between pH 6.3 and 7.5) would play a crucial role in the compound behavior under physiological conditions. At pH > 7.5, the studied drugs were deprotonated, which decreased their solubility in water. Notably, the studied drugs would show different solubilities under different physiological conditions: in the blood, pH 6.4; in the liver, pH 7.2; and in colon tissue, pH 7.5–8.0. Crucially, the pH of tumor cells is very often lower than 7.0; therefore, the solubilities of these compounds should be better in tumor cells than in normal cells. Furthermore, the electrochemical studies indicated the potential of studied compounds to the reduction as well as to the oxidation. Moreover, the reactivity of intermediate products resulted in the irreversibility of redox processes. This indicates the potential of enzymatic metabolites to be reactive with the following transformations to rather stable final products.

In the studies on phases I and II of drug metabolism, we aimed to determine whether (i) the metabolites of the studied compounds C-2028, C-2045 and C-2053 remain as dimers or undergo metabolic degradation into monomers and (ii) how therapeutic efficacy of these compounds would be influenced by their potential to metabolic transformations.

Studies with fractions of liver microsomes indicated that C-2028 was metabolized more quickly than C-2045 (Figure 3). ESI-MS analysis of the products generated during the incubation of the UAs with P4503A4 and P4502C19 isoenzymes pointed out that the metabolites of UAs generally retained their bisacridine dimer structure (Table 3) and both isoenzymes catalyzed the biotransformations to the identical products. The transformation rates observed in Figure 4 indicated that C-2028 and particularly C-2053 were metabolized significantly faster than C-2045 with both P450 isoforms. In conclusion, the presence of the hydroxyl group, as in C-2045, would protect the UAs against enzymatic degradation by cytochrome P450 isoenzymes. This result is unexpected considering the potential of hydroxyl groups to undergo enzymatic oxidation to alkoxyl radicals [64] and quinones [65]. However, these findings are in line with our previous results, which indicated that the hydroxyl group in the imidazoacridinone monomer C-1311 did not undergo metabolic oxidation in the presence of P450 isoenzymes [43,49,66].

The proposed metabolite structures observed selectively with FMO1 isoenzyme of phase I metabolism indicated the new pathway, which was not observed with P450 isoenzymes. The advanced step of UAs oxidation ran to N-oxides in the dimer-linking chains. Once more, there was not observed the oxidation with aromatic hydroxyl group. Therefore, the lack of reactivity at the hydroxyl group seems to be a unique feature of the 8-hydroxyimidazoacridinone in both monomer and dimer structures. This should be considered in the analysis of the relations between the activation of UA drug and/or its deactivation.

Our research on UGT-mediated metabolism of the studied bisacridines revealed the high selectivity of these transformations in relation to the compounds and to UGT isoenzymes (Table 4). Only one of the three studied drugs, C-2045, underwent glucuronidation and only the UGT1A family participated in this metabolism. The highest efficiencies in this family were seen with UGT1A1, UGT1A9 and UGT1A10 isoenzymes (Figure 8). Therefore, the product of drug glucuronidation is predicted to be found in the liver (UGT1A1) and kidney (UGT1A9) and extrahepatic metabolism in the gastrointestinal tract, particularly in colon tissue (UGT1A10), is also possible [43,44,45]. The glucuronidation products from the isoenzymes UGT1A1, UGT1A9 and UGT1A10 were identical and ESI-MS analysis identified an ion with *m*/*z* 792.25. This indicated that the dimer structure was maintained after the glucuronidation (Table 5). Because only C-2045 could be cleared by UGT-mediated metabolism, only this compound can be eliminated by the liver (UGT1A1) or by the colon (UGT1A10) immediately after drug delivery. As a consequence, intrinsic resistance to the mechanism of action of this drug would be observed.

The above results also showed that UGT-mediated mechanism of UA resistance is much less likely in the case of C-2028, which does not possess a hydroxyl group. Antitumor agents are very often susceptible to the deactivation via glucuronides; one example is irinotecan, which is applied as a first-line therapy for colorectal and gastric cancers [67,68]. The observed polymorphism of UGT1A1 is also responsible for the interindividual variabilities in belinostat antitumor treatment [69] as well as etoposide treatment [70]. Similarly, the extensive glucuronidation of epirubicin in the liver by UGT2B7 leads to drug excretion into the bile and urine and is a component of cancer cell resistance [71]. Therefore, in contrast to well-known antitumor agents, our lead UA, C-2028, expressed an additional promising and unusual therapeutic feature; specifically, it cannot be deactivated by the glucuronidation and, thus, it lacks intrinsic drug resistance. On the other hand, we have to consider other works, which reported on UGT conjugates of similar or significantly improved biological activity. This relates, for example, to morphine-6-glucuronide (650-fold-higher analgesic activity than morphine after UGT2B7 glucuronidation) [72,73] and to the glucuronidation of tamoxifen, an antitumor agent, by UGT1A4 [74,75,76,77]. We have also shown that the antitumor agent triazoloacridinone, C-1305, synthesized in our laboratory, expressed significantly higher cytotoxicity in UGT1A10-overexpressing KB-3 cells [63].

Several aspects were considered in the studies on the bisacridine compound C-2045 after its incubations with three tumor cell lines of different UGT expression and the following analysis of the culture media and cell extracts (Figure 9). First, the results with the HCT-116 cell line characterized by a low level of UGT isoenzymes indicated that the studied drug after cell penetration was in part excreted into the cell medium after 24 h and no metabolite products were observed. In the case of HCT-116 cells overexpressing UGT1A10, the compound was metabolized to the glucuronide, which was found only in the cell medium not in the cell extract. Further, in HT-29 tumor cells with natural high expression of UGT1A10, the high concentration of a glucuronide metabolite, G-C-2045, was found in both: the culture medium and cell extract. Moreover, a higher level of the native drug was found in the cell extract than in the incubation medium after a longer incubation period. This suggests that C-2045, the potent substrate of UGT1A10 isoenzyme, expressed high and/or specific affinity to some intracellular molecular target in HT-29 cells. Thus, the transfer of its native form outside the cell to the cell medium is not easy, in contrast to that observed for HCT-116 cells.

We also considered the significant inhibitory potential of two compounds C-2045 and C-2053 towards P450 and C-2028 towards UGT. The results presented in Figure 12 showed that the tested UAs inhibited the activity of P4503A4 and UGT isoenzymes, including UGT1A1 and UGT2B7. Therefore, the enzyme modulation potential induced by these compounds would influence metabolism of other therapeutics. For example, P4503A4 is responsible for the biotransformation of 45% of drugs undergoing modification by P450 [78]. In addition, UGT2B7 is responsible for 35% of drugs undergoing glucuronidation. One should also underline that the reduction of UGT1A1 activity would result in the disturbance of bilirubin natural metabolism in patients and would lead to hyperbilirubinemia [56].

Another aspect of the results described above is illustrated by Figure 9, Figure 10 and Figure 11. HPLC analysis indicated that HT-29 tumor cells, which are characterized by the highest levels of metabolic enzymes [79], are also the most susceptible to drug accumulation. This relates to all three studied compounds, but particularly to those possessing the additional methyl group in the acridine ring, C-2045 and C-2053. It cannot be excluded that this extra-susceptibility to accumulation is responsible for the observed high sensitivity of this type of colon cells to the unsymmetrical bisacridines, UAs, studied here [31].

## 4. Materials and Methods

### 4.1. Chemicals

Unsymmetrical bisacridines, UAs, C-2028, C-2045 and C-2053 were synthesized in the Department of Pharmaceutical Technology and Biochemistry, Gdańsk University of Technology, as described previously [29,30]. The following chemicals were obtained from Merck (Darmstadt, Germany): methanol (gradient grade for liquid chromatography), HCl, HEPES, alamethicin, NADPH and UDPGA. Ammonium formate was from Fisher Scientific (Pittsburgh, PA, USA). All other chemicals and solvents: H_3_BO_3_ (p.a. POCH, Poland), NaOH (p.a., POCH, Poland), NaCl (p.a., POCH, Poland) were of the highest purity available and were used as provided by manufacturers. Cell culture media McCoy’s 5A, antibiotics and trypsine-EDTA were obtained from Sigma Aldrich/Merck (Darmstadt, Germany), while fetal bovine serum (FBS) from Biowest (Nuaille, France).

### 4.2. Enzymes

Rat pooled liver microsomes, RLMs (20 mg of microsomal protein per mL), with untreated microsomal enzymes (UT-RLMs) and RLMs obtained after the treatment by P450 activity inducers: dexamethasone, Dex, 3-methylcholanthrene, 3MC and phenobarbital, PB, (P450-RLMs) were purchased from Sekisui XenoTech, LCC, Kansas City, KS, USA (through Tebu-Bio, Le Perray-en-Yvelines, France). The human recombinant P4502C19 and P4503A4 isoenzymes, co-expressed with human NADPH-cytochrome P450 oxidoreductase in *Escherichia coli* cells (Bactosomes), were from Cypex, Dundee, Scotland, UK (through Tebu-Bio, France). Human flavin containing monooxygenase isoenzymes, FMO1, FMO3 and FMO5 (55 mg of protein per mL) expressed in baculovirus-insect cells (Supersomes) were from Corning, NY, USA (through DIAG-MED, Warsaw, Poland). Recombinant human UGT1A1, 1A3, 1A4, 1A6, 1A9, 1A10 and UGT2B4, 2B7, 2B15, 2B17 (5 mg of protein per mL), expressed in baculovirus-insect cells (Supersomes), were purchased from Corning, NY, USA (through DIAG-MED, Poland). Each enzyme tested in this study is known to be active toward substrates specific for that isoform (7-ethoxycoumarin for RLMs, testosterone for P4503A4, imipramine for P4502C19, SN-38 (7-ethyl-10-hydroxycamptothecin) for UGT1A1, TFP (trifluoperazine dihydrochloride) for UGT1A4, epirubicin for UGT2B7 and TFK (7-hydroxy-4-(trifluorom- ethyl)coumarin) for other UGT isoforms.

### 4.3. PKa Value Determinations

The concentration of the compounds in the working solutions was 0.02 mM, pH was changed in the range 1.5–11 at 23 buffer solutions: 1.5–3.5 (interval 0.5 unit—HCl solution), 4.0–5.5 (interval 0.5 unit—acetic buffer [80]), 6.1–7.5 (interval 0.2 unit—PIPES (2-[4-(2-sulphoethyl)piperazine-1-ethanesulphonic acid) buffer [81]), 7.7–8.1 (interval 0.2 unit—HEPES (4-(2-hydroxyethyl)piperazine-1-ethanesulphonic acid) buffer [82]) and 9–11 (interval 1 unit—borate buffer [83]).

PKa values for tested UAs were determined by spectrophotometric titration using a Cary 300 Bio instrument. The UV-vis spectra obtained for all solutions in the examined pH range were subjected to the chemometric technique principal component analysis [67]. This procedure makes it possible to determine the number of spectral forms present in the tested samples and to estimate the mole fractions for each of them. To determine pKa values the relationships between the mole fractions of individual spectral forms and the pH of the samples were used. The first approximation of pKa values have been assigned to the values of pH corresponding to the central points of each titration wave. Numerical optimization by the adjustment of pKa values to the obtained molar fractions allowed to refine these values and the cross-validation procedure let to determine their standard deviations.

### 4.4. Voltammetric Analysis

Cyclic voltammetry (CV) and differential pulse voltammetry (DVP) were performed in 0.02 M borate buffer with 0.15 M NaCl, in water pH 7.4. with Autolab, model PGSTAT 12 potentiostat equipped with an ECD amplifier module (RC time settings: 0.0 s for scan rates > 10 mV/s and 0.1 s for scan rates < 10 mV/s). The voltammetric measurements were done in the three-electrode system consisting of the working electrode (glassy carbon electrode, GCE; *ϕ* = 3 mm; BAS Instruments), the reference electrode (Ag/AgCl/3 M KCl) and a platinum wire as the auxiliary electrode. Before each experiment the surface of working electrode was polished with 1 μm Al_2_O_3_ powder on a wet pad. After each polishing, to remove alumina completely from the electrode surface, the surface was rinsed with direct stream of ultrapure water. To minimize the electric noise all voltammetric experiments were performed in Faraday cage.

### 4.5. Metabolism with Rat Liver Microsomes and Recombinant P450 Isoforms

UT-RLMs (2 mg/mL) and P450-RLMs induced with Dex, 3MC, or PB and recombinant P4502C19 and P4503A4 (1 mg/mL) were assayed for activity toward tested UAs as follows. The proteins were incubated in a buffer containing 0.1 M HEPES (pH 7.4), 2 mM MgCl_2_ with 0.05 mM substrate in total volume 70 µL. Substrates were added also in buffer HEPES, pH 7.4. Reactions were started by the addition of NADPH (2 mM) and were incubated for specified time at 37 °C. The reactions were stopped by the addition of 8.75 µL of 1 M HCl, followed by centrifugation at 13,400 rpm for 10 min to pellet the denatured protein. The supernatant fractions were used for high-performance liquid chromatography (HPLC) analysis. Control reactions omitting substrate were run with each assay. All incubations were performed in two repetitions.

### 4.6. Transformations with Recombinant FMO Isoforms

Recombinant FMO1, FMO3 and FMO5 (0.5 mg/mL) were assayed for activity toward tested UAs as follows. The proteins were incubated in a buffer containing 0.1 M HEPES (pH 7.4), 2 mM MgCl_2_ with either 0.01 mM substrate in total volume 70 µL. Substrates were added also in buffer HEPES, pH 7.4. Reactions were started by the addition of NADPH (0.3 mM) and were incubated for specified time at 37 °C. The reactions were stopped by the addition of 8.75 µL of 1 M HCl, followed by the centrifugation at 13400 rpm for 10 min to pellet the denaturated protein. The supernatant fractions were used for HPLC-tandem with mass spectrometry (HPLC-MS) analysis. Control reactions omitting substrate were run with each assay. All incubations were performed in two repetitions.

### 4.7. Transformations with Recombinant UGT Isoforms

Recombinant UGT isoform protein (1 mg/mL) was assayed for activity toward tested UAs as follows. The proteins were incubated in a buffer containing 0.1 M HEPES (pH 7.4), 8 mM MgCl_2_ and alamethicin (25 μg/mL) with either 0.05 mM substrate in total volume 70 µL (alamethicin was added to permeabilize the membrane) [68]. Substrates were also in buffer HEPES, pH 7.4. Reactions were started by the addition of UDPGA (5 mM) and were incubated for specified time at 37 °C. The reactions were stopped by the addition of 8.75 µL of 1 M HCl, followed by centrifugation at 13400 rpm for 10 min to pellet the denatured protein. The supernatant fractions were used for HPLC analysis. Control reactions omitting substrate were run with each assay. All incubations were performed in two repetitions.

### 4.8. Drug Metabolism in Colon Tumor Cell Lines HCT-116 and HT-29

To analyze the transformation products of UAs in colon tumor cell lines, HCT-116 or HT-29 cells were plated in 60 mm Petri dishes; after reaching 80% of confluence, cells were treated with 5/25/50 μM of C-2028, C-2045 or C-2053 (prepared as 10 or 2 mM stock solutions in water) for specified time. Half of medium was added to acetonitrile (1:1, *v*/*v*), centrifuged (12 min, 13,400 rpm, 4 °C) and the supernatant was analyzed by HPLC (post-culture medium). Cells remaining on the Petri dishes were collected by gentle scraping, sedimented, washed with ice-cold PBS, suspended in ice-cold chloroform and lysed by sonication for 15 min. After 1 h on ice, insoluble material was removed by centrifugation at 13,000 rpm for 20 min at 4 °C. The sediment was discarded and supernatant (chloroform) were evaporated under pressure. Then, sample was suspended in ice-cold 60% methanol and directly analyzed by HPLC (cell extract).

### 4.9. Modulation of P4503A4 and UGT Activity In Vitro

Isoenzyme activity was studied by the rate measurement of β-hydroxylation of testosterone (specific substrate for P4503A4) and of glucuronidation of specific substrates, namely, SN-38 (UGT1A1), EPI (UGT2B7),) and TFK (UGT2B15). All substrate and drug solutions were prepared as 10 mM stock solutions in DMSO and were diluted in 0.1 mM HEPES (pH 7.4). The mixture containing isoenzymes P4503A4/UGT (0.5 mg/mL), probe substrate (0.1 mM), cofactor 2 mM NADPH/5 mM UDPGA and the increasing concentrations of tested UA (0, 0.01, 0.02 and 0.05 mM) was incubated in the reaction buffer containing (i) for P4503A4 0.1 M HEPES, 88 mM MgCl_2_ and (ii) for UGT 0.1 M HEPES, 88 mM MgCl_2_, 25 μg/mL alamethicin at 37 °C for 30 min and were terminated by adding 8.75 µL of 0.1 M HCl. The samples were placed in ice for 10 min and centrifuged (10 min, 13,400 rpm). Supernatant fractions were analyzed by HPLC. For more accurate investigation of P4503A4 inhibition by C-2045 and C-2053 the β-hydroxylation rate was measured at various concentrations of testosterone: 0.02, 0.05, 0.1 and 0.2 mM. The inhibitor (UA) concentrations were 0, 0.001, 0.005 and 0.01 mM, whereas the incubation time 30 min and concentration of P4503A4 (0.5 mg/mL).

### 4.10. HPLC-UV-vis Analysis—Products of Electrochemical and Metabolic Transformations

HPLC analyses of the described above the final supernatants obtained after electrochemical, enzymatic and cellular transformations were performed using a Nexera-I LC-2040C 3D HPLC system and the LabSolution software package (Shimadzu Corp., Kyoto, Japan). Samples were separated using a reversed-phase 5 μm Suplex pKb-100 analytical column (0.46 × 25 cm, C18) (Supelco, Bellefonte, PA, USA) warmed to 25 °C. The analyses were performed at a flow rate of 1 mL/min with the following mobile-phase system: a linear gradient from 15% to 80% methanol in ammonium formate buffer (0.05 M, pH 3.4) for 25 min, followed by linear gradient from 80% to 100% methanol in ammonium formate buffer for 3 min. The column was then re-equilibrated at initial conditions for 10 min between runs. The elution of each metabolite was monitored at 430 nm.

### 4.11. HPLC-ESI-MS, HPLC-ESI-MS/MS and HPLC-ESI-Q-TOF-MS Identification of the Transformation Products

HPLC-mass spectrometry analyses were carried out on a Nexera-I LC-2040C 3D HPLC system coupled to the LCMS-2020 system through an electrospray ionization (ESI) interface (Shimadzu Corp.). ESI-MS detection of the transformation products was performed out in the positive ion mode (+) in the full scan mode (mass-to-charge ratio, *m*/*z* 200–1000). HPLC-tandem mass spectrometry analyses in a product ion scan were performed with Agilent system 1290 Infinity II LC coupled to the 6470B system with a triple quadrupole in the positive ion mode (+), mass-to-charge ratio, *m*/*z* also 200–1000. HPLC-tandem mass spectrometry analyses were performed with an Agilent 6500 Series Accurate-Mass Quadrupole-Time of Flight (Q-TOF) mass spectrometer (Agilent Technologies, Santa Clara, CA, USA) controlled by Agilent MassHunter Workstation software. To ensure accurate mass during the experiment, the mass spectrometer was calibrated daily using calibration solution (ES-TOF reference mix, Agilent Technologies).

### 4.12. Statistical Analysis

The results are presented as mean ± SD of at least three independent experiments. One way analysis of variance (ANOVA) followed by student’s test were used. *p* values < 0.05 was considered statistically significant.

## 5. Conclusions

The studies presented above on drug metabolism improved by the investigations of their electrochemical conversion demonstrated rather low susceptibility of the studied unsymmetrical bisacridines to the electrochemical and phase I metabolic transformations. Moreover, our results indicated that the majority of metabolic products of the investigated agents with P450 and FMO enzymes retained their dimeric structure. The compound’s main metabolic pathways were the reduction of the nitro group in 1-nitroacridine ring of the dimer and the dealkylation of the nitrogen atom in the linking chain. The hydroxyl group in the imidazoacridinone ring is resistant to P450 and FMO metabolism. The specific phase II metabolic transformations took place with UGT isoenzymes, namely, UGT1A1, UGT1A9 and UGT1A10 and occurred in only one of the dimers, C-2045, which possesses the hydroxyl group. Moreover, in human colon HT-29 cells, we demonstrated the presence of an identical metabolite, which was identified as C-2045 glucuronide, G-C-2045. In respect to drug–drug interactions one should consider that the studied potent antitumor drugs influence the activity of the selected P450 and UGT isoenzymes.

Summing up, the results presented above allowed us for the detection of potent metabolites of the studied drugs including phase I and II drug transformations. This is the first step in the following prediction of therapeutic efficacy and drug resistance in future antitumor therapy with the potent UA drugs.

## Figures and Tables

**Table 1:** Chemical structures and individual background properties of the UAs studied.

Symbol	Structure	HPLC Retention Time [min]	UV-Vis Spectrum	ESI-MS *m*/*z*
C-2028	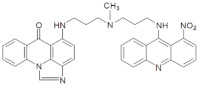	16.84	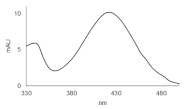	586.25 [M + H]^+^
C-2045	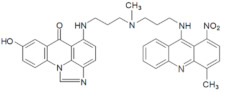	15.42	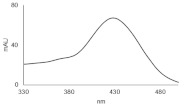	616.25 [M + H]^+^
C-2053	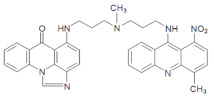	18.66	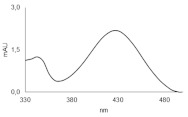	600.25 [M + H]^+^

**Table 2:** PKa values determined for the studied UAs. The standard deviations of the results were determined by cross-validation procedure.

Compound	pKa 1	Standard Deviation	pKa 2	Standard Deviation	pKa 3	Standard Deviation
C-2028	2.22	±0.01	6.01	±0.005	7.41	±0.02
C-2045	2.33	±0.007	6.37	±0.005	7.50	±0.02
C-2053	2.19	±0.01	6.19	±0.003	7.49	±0.01

**Table 3:** The structures proposed for main metabolites of UAs formed with P4503A4 and 450P2C19 isoenzymes (1 mg/mL P450 and 2 mM NADPH cofactor) on the basis of their ESI-MS and ESI-Q-TOF-MS (ElectroSpray Ionistaion-Quadrupole-Time of Flight-Mass Spectrometry) analyses.

Metabolite	HPLC Retention Time [min]	ESI-MS *m*/*z*	ESI-Q-TOF-MS *m*/*z*	Proposed Structure
M1^28^	15.06	556.25 [M + H-30]^+^	556.2799 [M+H-30]^+^	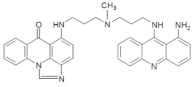
M2^28^	18.10	568.25 [M+H-18]^+^	568.2458 [M+H-18]^+^	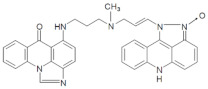
M3^28^	18.42	572.25 [M+H-14]^+^	572.2403 [M+H-14]^+^	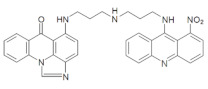
M4^28^	15.64	307.25	307.1545	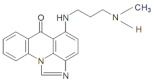
M1^45^	14.10	586.25 [M+H-30]^+^	586.2928 [M+H-30]^+^	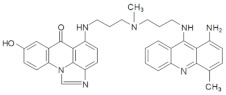
M2^45^	18.40	598.25 [M+H-18]^+^	598.2554 [M+H-18]^+^	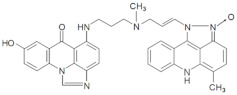
M1^53^	16.23	570.25 [M+H-30]^+^	570.2975 [M+H-30]^+^	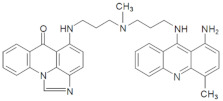
M2^53^	20.50	582.25 [M+H-18]^+^	582.2612 [M+H-18]^+^	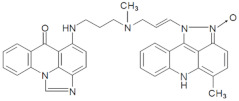
M3^53^	20.16	586.25 [M+H-14]^+^	586.2563 [M+H-14]^+^	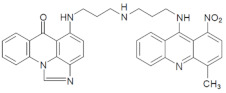

**Table 4:** Glucuronidation potency of the compounds C-2028, C-2045 and C-2053 (0.05 mM) with UGT1 and UGT2 families of human recombinant UDP-glucuronosyltransferases (1 mg/mL UGT and 5 mM UDPGA cofactor), Symbols “−“ and “+” means the lack of UGT activity and the significant UGT activity, respectively

	UGT 1A1	UGT 1A6	UGT 1A9	UGT 1A10	UGT 2B4	UGT 2B7	UGT 2B15	UGT 2B17
C-2028	−	−	−	−	−	−	−	−
C-2045	+	+/−	+	+	−	−	−	−
C-2053	−	−	−	−	−	−	−	−

**Table 5:** The structure of glucuronidation product obtained after C-2045 metabolism with human recombinant isoenzymes UGT1A1, UGT1A9 and UGT1A10.

Metabolite	HPLC Retention Time [min]	UV-vis Spectrum	ESI-MS *m*/*z*	Proposed Structure
G-C-2045	11.47	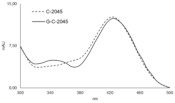	792.25 [M+H+176]^+^	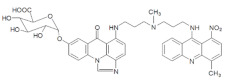

## Data Availability

Data available in a publicly accessible repository.
